# Hygiene of the Turkish Army

**Published:** 1857-07

**Authors:** J. N. Radcliffe

**Affiliations:** late of the Staff of His Highness Omar Pasha.


### ORIGINAL COMMUNICATIONS.

### THE HYGIENE OF THE TURKISH ARMY.

By J. N. RADCLIFFE, M.R.C.S.Eng., late of the Staff of His Highness Omar Pasha.

### I. THE SOLDIER.

CLAD in a dress which is not adapted to his habits, and equipped with somewhat cumbrous accoutrements, the aspect of the Turkish soldier is not, at the first glance, very satisfactory. While the dark regulation blue of his uniform retains its pristine tint, and the bright red of his fez is yet undimmed, and while his weapons still glitter with the polish of the manufactory, he has some semblance of that neatness which the occidental looks for in the well trained soldier. But when, after a brief period, the short-cut tunic has shrunk out of all proportion, and has become flecked with brown and greyish-white stains, as of mildew, the original blue having early faded; when the coarse strands of the cloth are visible at many a roughly used point; when the fez has become pale or clouded, and the tassel, almost devoid of colour, hangs dishevelled beneath the dimmed brass;* when the ungainly slippers look still more ungainly from engrained dirt, which is never removed by the brush, or hidden by blacking; when the weapons and accoutrements bear glaring marks of imperfect cleansing; and when, together with much that is untidy, there is an absence of all smartness, then the Turkish soldier seems but a caricature of western military fashions.

This, indeed, was the general aspect of the Turkish soldier during the war of 1854-6; but notwithstanding that his slovenly and unsoldier-like bearing might rout every preconceived notion of military requirements, yet the most superficial observer could not fail to notice the matured physical powers of the man, and the ease with which he moved under a heavy pack. When the eye had become accustomed to the shortcomings of dress and bearing, it soon recognised the admirable stuff of which the Turkish soldier was made, and his fitness for a military life.

Temperate in his habits, the duties of the barrack and of the camp deprive the Turkish soldier of no luxuries that he cares for; accustomed to a diet admirably adapted, from its sim-

* The soldiers of the Turkish army, and the sailors of the navy, have a small circular plate of polished brass fixed upon the summit of the fez. This plate is a distinctive mark of the Imperial military and naval service.

plicity, for the field, and having few wants, he can struggle with the vicissitudes and hardships of a harassing campaign, perhaps better, and with less suffering, than the soldiers of any other nation; and, as he has few vices, the restraint of military discipline does not fret him. He is not, as a rule, cleanly in his person, unless he can have access to a bath, which he will use as often as his means will permit; or unless he is under the command of a smart officer. The soldier may certainly perform with scrupulous regularity the daily ablutions prescribed by his religion; but these ablutions, as ordinarily practised, are not such as to ensure cleanliness. He is indolent, and ignorant of most things beyond the immediate sphere of his military and religious duties. Steady in the former, he is regular in the latter; and, during a campaign, he may be seen, at the appointed hours, within the camp, upon its verge, or at the road-side, performing the accustomed prostrations, and repeating the necessary prayers; and at sunset, whole battalions and regiments, headed by their priests, will draw up in front of their encampments, and, with all the regularity and order of a parade, fulfil their devotions.

The most marked characteristics of the Turkish soldier during the war of 1854-6, were maturity of age and of physical development; and in these respects, so far as could be judged by a general examination, in the absence of all numerical data, he contrasted most favourably with the soldiers of the other sections of the allied forces, and particularly with the British and French soldiers.

The influence of matured physical powers in maintaining the efficiency of an army in the field, particularly during a harassing campaign, is an important question of military hygiene. The experience of our own army in the Crimea, would seem to show most conclusively that immaturity of age, and incomplete physical development of a considerable portion of the men, were among the most potent predisposing causes of disease; and, also, that they were influential in fostering a high degree of mortality. That the matured physical powers of a great proportion of the Turkish soldiers had no inconsiderable effect in maintaining the efficiency of the Turkish forces during the Crimean campaign, notwithstanding the protracted sufferings entailed by a wretched *quasi* Commissariat,* and the bad hygienic condition of crowded and long occupied camps, may be fairly assumed.

Dr. Lyons, in his *Report on the Pathology of the Dis-*

* The Turkish army has no regular commissariat.

*eases of the Army in the East,* makes the following instructive remarks on extreme youth and incomplete physical development as a predisposing cause of disease amongst the troops:—"In treating of the various predisposing causes of disease in operation amongst Her Majesty's forces in the East, there is one which may be classed amongst physiological influences, and which demands most earnest consideration at your lordship's hands. I allude to the extreme youth, incomplete physical development, and general immaturity of a very considerable proportion of the strength of several regiments; constituting, as I have reason to believe, in the total of the army, a number sufficiently great to render the question one of vital moment and of even national interest, as largely affecting what may be called the physiological economy of the army. Amongst even well matured constitutions, the hardships and fatigues, trials, privations, and exposure of campaigns, such as those of the past years in the Crimea, must almost of necessity prove largely productive of disease, and induce much mortality. But on the undeveloped frames, and the unripe strength of the ill seasoned recruit, such causes operated with two-fold energy, and with a more than doubly fatal effect. Not only does the power of resistance to morbid influences, and the consequent invasion of disease in such youths, seem inferior to that of the older soldier, but when attacked they succumb more readily under the effects of a fever or a flux. And even when such a disease does not prove immediately fatal, the convalescence of the young is generally slow, constantly attended by a disposition to relapse, or to the development of those secondary enteric lesions, the almost unfailing result of such fevers as those which prevailed amongst the troops of the East. * * * The results of surgical pathology corroborate, to a large extent, the observations just made, which are chiefly based on considerations arising out of the behaviours of the immature constitution under the effects of disease. As a general rule, true, at least, in a very large part of its usual acceptance, youth forms a favourable element in the calculation of chances of surgical cases. Amongst the troops in the Crimea, however, no such favourable anticipations could be indulged in on the score of youth. The constitution of the young, even independently of the presence of actual disease, seems much impressed by the influence of the various causes dwelt on; many succumbed almost immediately under the shock of injuries; and in the case of the graver surgical operations, no more advantageous results, but the contrary, appeared to be shown on the side of the young: in fact, youth was not to be counted on as a favourable element in determining a prognosis in such cases." (p. vii.)

The Turkish army is recruited by voluntary enlistment, and by a species of conscription among young men of twenty years of age, and upwards, or among those who are supposed to have reached that age. Every man pronounced to be in good health and of the proper age, is summoned to form part of the annual contingent, but only one member of each family is enrolled, and only sons are exempt. The conscription falls upon the lower orders, the rich escaping. The officers, as a rule, are obtained from the same class of society as the men, and, in consequence, they are extremely ignorant. It is estimated that the male population of Turkey in 1844 amounted to 19,533,124, of which 4,784,490 were of military age (twenty to forty years); a levy of one man in ten of the military age would give a force of 478,449 men. (*Registrar General's Sixteenth Annual Report*, p. 122.)

### II. THE DRESS.

The uniform of the Turkish soldier is made of the coarsest materials, and its colour, dark blue, is fleeting. Moreover, the regulation tunic has a wretched and uncomfortable aspect, and straight-cut trousers are unsuited to habits of kneeling and sitting cross-legged. The soldier is not ill clad, so far as amount of clothing is concerned, and his dress is in several respects well fitted to the climate of the different parts of the Turkish empire. The complete suit of strong cotton which every man wears next the skin, is calculated to protect the surface from those slight, but sudden, alterations of temperature which not unfrequently accompany exposure to the evening breezes, or the light airs of early morning, during the hotter seasons of the year, and which are apt to induce serious functional and other disorders. On the other hand, during the colder seasons, the under-clothing, which is then made of cotton of a closer and heavier texture, contributes in an important manner to the maintenance of the warmth of the body.

In the summer, loose white trousers are worn, and, according to the regulations of the service, a white jacket should be issued to each man; and when, at this season, (as in the case of the Egyptian troops,) the men are clad altogether in white, the effect is most pleasing, particularly on parade. The loose white dress sets off both the men and the accoutrements, and the bright red of the fez, and its polished brass plate, give a brilliant finish.

The fez affords slight protection against rain and the sun, and it does not give any shade or defence to the eyes; yet, obnoxious as this head-dress may be to western notions, no very apparent evils arise from its use—at least, trustworthy evidence of its harmfulness is wanting. The hood, which is attached to every soldier's over-coat, is an additional protection to the head from wet; the existence of any peculiar tendency to evil effects arising from undue action of the sun's rays upon the head, and consequent upon the use of the fez, is doubtful; and although some medical men consider that the fez induces an unusual amount of ophthalmic affections amongst the Turkish troops, yet these affections are so frequently, if not generally, associated with filthy habits, that the correctness of the conclusion may be doubted.

Early in the spring of 1855, when attached to the Turkish forces at Eupatoria, I noticed that a large number of the soldiers were affected with *ophthalmia tarsi*. The disease was almost invariably connected with great personal filth, and it was most commonly found among those men who were crowded in the filthy hovels within the town, particularly in the deserted shops, which had never been fitted up as dwelling-houses. In these wretched quarters the men sought warmth from close crowding and incandescent charcoal, and they rested upon floors of hardened mud. At the same time the food of the men was insufficient and of bad quality, and the water chiefly used was brackish.

The infantry are shod with loose strong slippers; the cavalry with boots which extend to the middle of the leg; and both classes of soldiers wear thick woollen stockings.

The soldier loves to have his legs warmly clad, and he often has recourse to sundry effective but ungainly expedients, in addition to the means provided for him, to attain this end. The peasant recruit, accustomed to enwrap his legs in some thick material, the folds of which are kept in their place by ligatures of twine, carries this habit into the camp, and the suburban or town-bred recruit adopts the more sightly "legging" of eastern costume. It is not uncommon, during a march, to see the soldier with his trousers and drawers rolled up to the knee, exposing legs so swathed as to be almost shapeless.

The slipper is, perhaps, the most objectionable portion of the soldier's dress. An abomination in wet weather, and a serious impediment in mud, it is only when the ground is hard and dry that this form of covering for the feet is at all supportable. Certainly from habit the Turk shuffles along exceedingly well in the slipper, and it is endeared to him by the usages of his religion; but thus shod the soldier can rarely, if ever, acquire that smartness of action, and precision of step, which is requisite to the perfection of a western drill. In the best trained regiments, shortcomings from this source may be discerned, and no cause contributes more to that want of a soldier-like bearing, which is so conspicuous among the Turkish troops.

The slipper is an imperfect protection against wet and damp, and its use is doubtless a common source of disease, although habit, and the custom which is usually practised by the Turkish soldier, particularly in inclement weather, of clothing the legs warmly, may diminish the chances of mischief.

Each soldier is provided with a long large over-coat, to which is attached a capacious hood. The over-coat, which is of sufficient length to extend below the middle of the leg, is made of coarse, heavy cloth, and it shields the body tolerably well from both wind and rain.

The Turk has a habit which is very instructive. When in the morning or the evening, during the hotter seasons, he reclines in the open air, or before the unclosed window, he will generally have cast loosely around him, if he be of the wealthier class, a large coat lined with fur, or if he be of the poorer class, a heavy sheep-skin coat. Thus, without incommoding himself, he is safely protected from the evils which the cooler hours of the day might bring to him, and he resists more effectually the insidious poison which is often borne upon the wind. For in most parts of the Turkish empire the wind, sweeping over imperfectly cultivated or marshy tracts of land, is apt to be laden with pernicious malaria, which finds a lodgment most readily during the hours when the system is relaxed.

Another habit customary with the Turk when enjoying himself in the open air, and worthy of note, is the use of the carpet spread upon the ground. The carpet not only makes a cleanly seat, but it also protects from whatever moisture there may be at the surface of the earth.

The Turkish soldier practises both these habits, and he may be seen, in the intervals of duty, squatted upon his carpet, and with his large, hooded great-coat thrown loosely over his shoulders, enjoying the cooling breeze, and inhaling the grateful fume of tobacco.

The issue of clothing to each soldier is as follows:—One uniform coat, one pair of uniform trousers, two pairs of white trousers, a light jacket for summer, two winter shirts, two summer shirts, two pairs of winter drawers, two pairs of summer drawers, two pairs of stockings, and one fez, every year; one pair of slippers every three months ; and a great-coat every three years. A carpet, measuring about six and a half feet in length and four feet in breadth, is also issued to each man yearly.

The weight of the knapsack and accoutrements of the infantry soldier, in marching order, and exclusive of the weapons, is full twenty *okes* (fifty-five pounds).

### III. THE FOOD.

If there be one subject in connection with military hygiene. which is more important than another, it is that of food ; for the efficiency of an army is, perhaps, more dependent upon the character of its food than upon any other thing.

The food of the Turkish soldier is in many respects singularly well adapted to a military life. At first the coarse brown bread, the dark hard biscuit, the lean ill fed meat, the lard-like fat, and the filthy rice, which form the bulk of the rations, have a repulsive aspect to the occidental, and they seem ill calculated for the proper nutrition of the soldier ; but the food is such as he has been habituated to from childhood ; the bread, when made with ordinary care, is sweet and wholesome ; a substitute for the scanty fat of the meat is furnished by the *yagh*—the sheep's tail fat—of the rations ; and dirt is a remediable and the only evil of the rice.

The daily ration scale of the Turkish soldier is as follows :—*Bread,* three hundred drachms (*i. e.,* about thirty-four ounces : eight drachms and three-quarters being equal to one ounce avoirdupois) ; *meat,* ninety-two drachms (ten ounces and a half), except on two days of the week, when no meat is issued ; *rice,* twenty-five drachms (two ounces and three-quarters), which are used in the preparation of soup ; but on the days when the soldier receives no meat, ninety-two drachms (ten ounces and a half) of rice are issued for the preparation of *pillaff ; yagh**—sheep's tail fat—sixteen drachms (one ounce and three-quarters) ; *olive oil,* twenty-five drachms (two ounces and three-quarters), used for light and for cleaning arms, as well as for food ; *salt,* about four drachms (half an ounce) ; *vegetables,* (onions, *nohud* (chick-pea ?), haricot-beans, etc.) a variable quantity.

The issue of rations to the Turkish soldiers is upon the same scale, and includes all the before-mentioned articles of food, in peace and in war : and in this respect the regulations of the Turkish army differ materially from those of our own

* "*Yagh*" is a general term for butter, grease, tallow, oil, fat, etc.; but the "*yagh*" of the soldier's rations is generally prepared sheep's tail fat.

army. In the British army, under ordinary circumstances, and in non-tropical climates, bread, or biscuit, and meat, are alone furnished to the men, the remaining articles of food being purchased by the soldiers themselves, subject simply to the control arising from the organisation of the messes.

The regulation governing the issue of rations in the Turkish army, although originating, in all probability, from another and far different source, is worthy of consideration in reference to hygiene.

It is a questionable policy which does not at all times provide for the soldier the full amount of those articles of diet which are requisite for the preservation of health; for in a matter of such importance it ought not to be left to the soldier, as in our own army, to eke out somewhat scanty rations from a scantier pay, as, notwithstanding that messing may obviate any serious mischief from this source, and that the men may prefer to purchase food for themselves (probably because they can thus obtain a more varied diet), yet a liberal ration scale, such as is adopted in our navy, would give the soldier a better regulated and sufficiently varied diet, and probably be more conducive to health.

Again, without the adoption of such a ration scale it would, perhaps, be impracticable to teach the soldier efficiently that knowledge of the best methods of preparing food, the want of which, in our own army, is not only a glaring evil, but it is also one of the gravest sources of disease during a campaign.

Moreover, unless a complete and constant ration scale be adopted, it is improbable that a class of officers can be trained who will be capable of contending properly with the difficulties of providing an army in the field with food. A knowledge of the character and value of food, and of the readiest substitutes for important articles of diet, cannot be obtained without a specific training, and it is from the want of this training that our Commissariat has been generally so lamentably inefficient at the commencement of a campaign. The sad illustrations in confirmation of this statement, which may be derived from the history of the Crimean war, will be still fresh in the memory.

The rations of the Turkish soldier are very liberal. He wants for none of the essentials of diet, and the proportion of meat, although small, is quite consistent with his habits and taste, meat being held in much less regard by the oriental than by the occidental. The rations, irrespective of vegetables, contain somewhat more than forty-nine ounces of solid aliment. This amount of food is more than the soldier requires under ordinary circumstances, and it is amply sufficient to satisfy his wants during a campaign.

Probably the soldier of no other nation in Europe is so plentifully rationed as the Turkish soldier, and the contrast with the rations of our own soldiers is great. The daily rations of the British soldier, in non-tropical climates, are one pound of bread, or three-quarters of a pound of biscuit, and one pound of fresh or salt beef, or pork. In tropical climates the rations are the same, "except that on two days of the week the issue of salt pork is reduced to twelve ounces, and the soldier receives in lieu of the remainder a daily issue of five-sevenths of an ounce of cocoa, one ounce and two-sevenths of sugar, and a quarter of a pound of rice, or half a pound of peas." (*Statistical Reports on the Sickness, Mortality, and Invaliding among the Troops in the United Kingdom, etc.*, 1853, p. 118.) Under extraordinary circumstances the amount of the rations is increased; and during the Crimean war the soldier received daily one pound and a half of bread, or one pound of biscuit, one pound of fresh or salt meat, two ounces of rice, and rations of sugar, and coffee, or tea. The usual daily ration of spirit, half a gill, was also increased to one gill. Thus the weight of the British soldier's rations, exclusive of vegetables, varies, under ordinary circumstances, from thirty-two to thirty-six ounces, and under extraordinary circumstances from thirty-four to forty-two ounces; in the first instance the amount of solid food being from thirteen to seventeen ounces less than in the rations of the Turkish soldiers; in the second instance from seven to fifteen ounces less. The common use of sugar, coffee, or tea, and spirits by the British soldier is not, however, to be lost sight of; and a regulation exists in our army by which the different messes may claim an extra amount of bread or biscuit if it be required. Moreover, the food of the British soldier is of a much better quality than that of the Turkish soldier.

The daily rations of the British sailor may be regarded as a standard of the amount of food requisite to maintain the body in full health. These rations contain from forty to forty-eight ounces of solid nutriment, inclusive of eight ounces of vegetables.

The Turkish soldier eats two meals daily, the first meal being taken two hours after sunrise, the second one hour before sunset, and in the interval the soldier rarely takes any food. The evening meal is the most substantial meal of the day. The rations of the Turkish soldier are amply sufficient for two excellent meals daily, and, if requisite, for a third and slighter meal.

It is difficult to dogmatise upon the times and the method in which food should be taken, as so much depends upon peculiarities of duty and of habit; but it is questionable whether a more fitting plan could be adopted than that which is used by the Turkish soldier, whether the subject be considered physiologically or in reference to the duties of a military life. The division of the rations into two full and substantial meals, and the consumption of these meals at the commencement and termination of the day, fulfil the most important physiological requirements for the sustentation of the body; for by such an arrangement the food is very equally divided over the whole day, and the interval which elapses between the meals admits of digestion and assimilation being fully and properly effected. Moreover, a solid and substantial meal at the commencement of the day is the best preparation for military duty, particularly in the field; and a still more substantial meal at the termination of the day is the method best adapted for recruiting the exhausted physical powers, and for preparing the soldier to undergo the fatigue of night duty.

The importance of the latter consideration during a campaign cannot well be exaggerated; and the experience of the Crimean war showed that the want of an arrangement by which the soldiers of our army could have a substantial evening meal, was equivalent to an actual deprivation of food.

The British soldier takes three meals daily. The morning and evening meals, consisting mainly of farinaceous substances with liquids, are comparatively slight; the noon meal is the principal meal of the day.

This arrangement, so long as the soldier purchases the greater portion of the articles of food which he consumes, and is not confined to a limited quantity, may act well enough; but when he is dependent upon rations alone, as during a campaign, the case is different. For the arrangement of the meals is incompatible with a true economy of the rations, the more nutritious portions of the food not being sufficiently distributed throughout the different meals; the morning and evening meals are not substantial enough for the physical requirements of a campaign; and the amount of the rations, so long as the system of cooking commonly pursued in the army is continued, is not sufficient to furnish the soldier with three ample meals daily. Hence it was found necessary to augment the ration scale of our army during the Crimean war.

It would be well if our soldiers were taught so to arrange their food, that the morning and evening meals should be the most substantial meals of the day. Such an arrangement, while it would enable the soldier to bear up better against the physical hardships of a campaign, would also contribute to the maintenance of a higher degree of health in the field, and many sufferings would in consequence be avoided.

The morning meal of the Turkish soldier consists of bread, and of a soup composed of rice and *yagh* seasoned with salt; the evening meal consists of bread, and of a stew formed of meat, rice, *yagh*, and vegetables, when the latter are issued. Twice in the week, when no meat is issued, *pillaff* forms the principal portion of the evening meal. Pillaff is rice cooked with fat, or butter. The rice having been boiled in water until softened, the superfluous fluid is poured off, and the grains are fully distended by exposure to a gentle heat. Then boiling fat, or butter, is poured over the rice, and mixed with it. The proportion of fat or butter in a pillaff should be sufficient to sheath each particle of rice; but the soldier, and the lower order of Turks generally, prefer the fatty matter in excess.

Well made pillaff is a satisfactory and substantial dish; and when richly flavoured soup is substituted for water as the agent for softening and distending the rice, the dish is most appetising. Pillaff, moreover, in the absence, or with a scanty supply, of meat, is not a bad campaigning dish. Its bulkiness is well fitted to satisfy the cravings of the appetite; the simplicity of its constitution is such that it may be readily prepared under most circumstances; and the materials of which it is composed are easy of carriage.

The bread used in the Turkish army is, when well made, sweet and palatable, although coarse, and, when practicable, it is issued freshly baked to the men every day. The ration-biscuit is exceedingly hard, but it is made of a finer and better flour than the bread. Before eating the biscuit, it is customary to break it into small pieces, and soften them by soaking in water, subsequently placing the fragments between a fold of damp cloth, or linen, and allowing them to swell.

When bread cannot be obtained, a quarter of an oke (eight ounces and a half) of flour is sometimes issued in lieu of biscuit. The men prefer the flour to the biscuit, as they can make a form of bread which is more grateful to the taste than biscuit. The usual method of preparing flour for food is very primitive, but it is suited for campaigning under circumstances when a supply of fresh bread cannot be obtained. The flour is mixed with water so as to form a paste of slight consistency. A portion of the paste is then spread out thinly upon an iron plate, and baked over embers. The cake thus made is termed

*ufka :* its taste is not unpleasant, and its appearance is similar to the thin oaten-cake of our northern counties, but the colour is darker.*

The Turkish soldier usually stews his meat, but occasionally he cooks it in a different fashion. One evening in June 1855, when the troops to which I was attached had bivouacked on the eastern bank of the Tchernaya, two men, from a battalion of Turkish rifles that had halted nigh at hand, approached a fire near to the spot where my tent was pitched, and prepared their evening meal. Each man had cut a small branch from a neighbouring bush, and this branch was first peeled, and cut to a point at one extremity. One of the men then drew from his haversack a portion of the intestines, and the other man produced a piece of the flesh, of a newly killed sheep. The soldier who possessed the intestine wrapped it, fold upon fold, round the branch he held in his hand, transfixing with the pointed extremity the last fold ; and the other soldier having cut his meat into small pieces, pierced them with the sharp end of his branch, and fixed them one after the other upon it. A little salt was then sprinkled over the meat thus spitted, and one of the men even added some pepper, which, after much fumbling, he produced from a small paper parcel. The spits were then held, in the hand, over the fire until the meat was cooked, when the intestine was disengaged mouthful by mouthful, and the fragments of mutton were drawn one by one off the spit ; and, with the addition of a large allowance of bread, each man ate his evening meal with avidity, and terminated it with copious draughts of water.

When Agamemnon feasted the " elder chiefs of all the Greeks, having pierced the entrails with spits, they held them over the fire. But then after the thighs were roasted, and they had tasted the entrails, they cut the rest of them into small pieces, and fixed them on spits, and roasted them skilfully, and drew them all off the spits." (*Iliad,* b. ii.) : and when Nestor feasted Telemachus, and " the thighs were burnt and they had tasted the entrails, they cut the other parts into bits and fixed them on the spits, and roasted them, holding in their hands the sharp spits." (*Odyssey,* b. iii.)

---

* Froissart, writing of the manner in which the Scots carried on war in the time of Edward III, states that, " Under the flaps of his saddle, each man carries a broad plate of metal ; behind the saddle, a little bag of oatmeal : when they have eaten too much of the sodden flesh, and their stomach appears weak and empty, they place this plate over the fire, mix with water their oatmeal, and when the plate is heated, they put a little of the paste upon it, and make a thin cake like a cracknel or biscuit, which they eat to warm their stomachs." (Vol. i, chap. xvii.)

Salted meat was not used in the Turkish army previous to the last war, when pickled mutton was from time to time issued to the soldiers. This mutton was exceedingly tough, and long stewing was required to soften it. Beef cooked in its own fat, with the addition of a little water, and kept in jars, was also occasionally issued. This form of preserved meat is termed *kavourma,* and it is a better and more palatable article of food than pickled mutton.

The cooking utensils used by the Turkish soldier consist of a circular thin iron plate, and a broad, shallow iron vessel, which is tinned within. One of each of these utensils is issued to every ten men.

In the preparation of a limited quantity of food, such a system of cooking should be adopted as will economise the food most, secure the whole of its nutritive properties, and adapt it best for the action of the digestive organs. These requisites are fully attained by the method of cooking commonly used by the Turkish soldier; and the system of stewing which he ordinarily pursues in the preparation of his food is that by which such requisities can be most readily fulfilled. The stew-pan wastes nothing, loses nothing, and its capacity is illimitable; moreover, no other cooking vessel can be so easily manipulated.

The Turkish soldier, inasmuch as the stew-pan is, as it were, his national cooking vessel, has an advantage over the English soldier. The English soldier is habituated mainly to roast or boiled joints, and to the preparation of vegetables in a simple and separate form, and no method of cooking is less calculated to economise his rations, or is more unfitted for the field. Indeed, if the rations of each soldier were issued singly, they would prove, as a rule, insufficient for the ordinary consumption of the man, so long as the method of cooking usually adopted in the army is persisted in, and it is only by the system of messing that such a shortcoming is avoided.

The evil results of a vicious system of cooking were very palpable among our soldiers during the last war. Their food was too commonly presented to the stomach in a form ill adapted for digestion, (considerable discomfort and suffering, if not actual disease, arising from this source alone), and the rations were partly wasted. The hard masses of badly boiled, roasted, or grilled meat, and of worse cooked vegetables, were most pernicious while the soldiers were exposed to the influence of those powerful miasmatic emanations which were peculiarly rife in the camp, and which predisposed in an especial manner to intestinal affections.* It was a common observation among the healthiest soldiers, that boiled vegetables almost invariably passed through the intestines and appeared in the ejections nearly unaltered; and upon this ground alone the medical officers of some divisions, during the summer of 1855, advised the discontinuance of the ration of potatoes, lest the imperfectly digested vegetable, in its passage through the bowels, should excite diarrhœa or dysentery. Had the vegetable been cooked in such a fashion that it would have been reduced almost to a pulp, probably complete digestion might have been ensured, and the nutritive effects of this important article of diet might have been fully secured, while the evils resulting from the presence of crude vegetable matter in the digestive tract would have been avoided. A portion of the rations was lost, because the method of cooking necessitated a certain amount of waste, and the ration of one valuable article of food could not generally be made use of in a manner satisfactory to the palate. In several divisions the ration of rice was rarely drawn by the men, for they were not acquainted with a method of cooking which would give to it a grateful savour; and this valuable aid to the contents of the stew-pan (in which, while adding to the bulk of the food, the rice receives the flavour of the more sapid contents) was lost. Thanks to M. Soyer, some portions of the army, before the

* "Of the various causes operating in the production of disease amongst the troops in camp, the greater part tended undoubtedly to the development of morbid action in one part of the system more especially, and this almost to the exclusion of affections in other organs, at least in a primary form. The abdominal viscera were those in which disease was most commonly manifested. Physical causes from without, and irritation established within, in some measure, at least, connected with peculiarities of life and diet, led to a predominant tendency to gastro-enteric derangements, and the consequent development of the diseases known as fluxes, in their various forms. It may, indeed, be said, that the main features of the pathology of disease, as presented in the army of the East, were embraced in the two classes of the fevers and the fluxes; and of the latter, no inconsiderable portion owed their origin to the former. Ample proofs will be found, in the various following sections of this Report, of the extraordinary predominance of abdominal lesions. To such an extent did they prevail, that I believe I shall be warranted in stating, that in no case submitted to examination by me or my assistant has a perfectly normal state of the digestive viscera been found to exist; while a marked absence of lesions in other parts was, in those who died in the early periods of disease or injury, the general rule. . . . . In no instance, in which a *post mortem* examination has been made by us, have the abdominal organs been found entirely free from disease. And these observations will hold good, not only in the case of those dying from acute or chronic disease, in which symptoms referrible to the abdomen were present, in a more or less marked degree, but also to all those cases in which death was the result of the shock of great mechanical injuries, or followed at an earlier period after the greater surgical operations." (Dr. Lyons' Report on the Pathology of the Diseases of the Army in the East, pp. xii, and 11.)

termination of the war, were taught better and more economical habits of preparing their food; but, owing to apathy on the part of the officers, and to a degree of obstinacy on the part of the soldiers, the good results of M. Soyer's instructions were but partial. Indeed, the dishes habitual to the soldier were not to be done away with during a brief campaign, and the field is not the proper place for tuition. Nothing is more essential to the maintenance of the soldier's health than a proper system of cooking; nothing is more important than a system which will enable him to husband his rations in the field, and add to them by any article of food which may chance to come to hand. The stew-pan is the only means by which such an end can be readily attained; but the use of the stew-pan and its capabilities, and a taste for the dishes which can be prepared in it, must be taught systematically to the soldier in times of peace. The soldier should be drilled in the mysteries of the stew-pan as well as in his manual exercise.

Water is the chief drink of the Turkish soldier, and he consumes large quantities of it; but, not unfrequently, he shows considerable indifference to its quality. In the field, his inattention to the precautions necessary for the protection of the springs from which he drew water, often led to their pollution. The Turkish hospital, at Balaklava, was supplied with water from a spring about two hundred yards distant from the hospital. At first the hospital attendants and guard drew water, along with the British soldiers stationed in the vicinity, from a spring at a greater distance from the hospital, but more easy of access. It was discovered, however, that the Turkish soldiers were in the habit of washing their cooking utensils, and even cleansing their foul linen, in the water at the spring-head, whereupon, a British sentry was placed near the spring, and the Turkish soldiers were forbidden to draw water from it. They had recourse then to a well from which a copious supply of excellent water could at all times be obtained, and which was nearer to the hospital. No control being exercised over the Turkish soldiers at this well, the water became in a short time irretrievably polluted. The men washed their linen, and cast out water, unnecessarily, upon the brink of the well, and the ground in the immediate vicinity was converted into a deep slush, which oozed through the interstices of the stone-work, and trickled into the water. No attempt was made to remedy the evil, and at last the water became offensive even to the smell. Yet this was the only source from which the supply of water for the hospital was drawn for several months, notwithstanding that two or three small unused springs existed on the plateau near the hospital. Once, indeed, when the water had become highly offensive, an attempt was made to discover a spring which had existed very near the hospital, but which had been lost in consequence of the formation of a burial-ground, belonging to certain of the British forces on the heights. An exploration was commenced near the lower border of the burial-ground; but, fortunately, the spring was not discovered, for I subsequently ascertained that the water, diverted from its original course, permeated the graves, and traversed the burial-ground near its centre.

Even the springs contiguous to the head-quarters encampment, and immediately under the eye of the commander-in-chief, were ruined by the filthy habits of the soldiers, and it was necessary for the men to procure water from wells half-a-mile distant from the tents. The banks of a copious spring, in a hollow on the verge of the encampment, was used as a latrine, and a depository for carrion.

Spirit-drinking is so rare a vice among the Turkish soldiers, as scarcely to merit a passing notice; but the officers are not so free from the pernicious habit, and with them it assumes its worst form, the spirit (of whatever kind it may be) being drunk pure, and often in large quantities.

Coffee and tobacco are the two great luxuries of the soldier, but he has to provide them from his own resources. The coffee is used after the fashion of a cordial. It is prepared, generally from the freshly ground and recently roasted berry, as a strong decoction, of which the dregs form one-third or one-half of the whole bulk. A quantity of this decoction, equal to about one ounce, (without either sugar or milk,) is commonly taken at one time, and the dregs are swallowed along with the liquid. This is the usual mode of drinking coffee in Turkey.* Tobacco is used according to the fancy of the individual; but the soldier often passes long intervals without it in the field, and he suffers much less discomfort from the deprivation than would be ordinarily supposed.

The Turkish soldier has great tact in economising his fuel—a tact arising from habit, wood and charcoal being almost the only kinds of fuel used in the Turkish empire. The Turk

---

* The introduction to Dr. Wagtail's disquisition on the verb *drink* may be fitly applied to the Turkish method of taking coffee. "The verb *drink*", as the learned doctor affirmed, "was improperly applied to the taking of coffee, inasmuch as people do not drink, but sip, or sipple, that liquor; .... the genuine meaning of drinking is to quench one's thirst." (Roderick Random, chap. xlv.)

rarely uses a fierce heat in cooking, and, over a handful of embers, he will perfect excellent dishes. The process of cooking with a gentle heat may require more time, but not so much as to militate against the advantages to be derived from the better method of preparing the food. A hole, about six inches in length, and five in breadth, formed like a horse-shoe, and dug into the side of a bank, or an enclosure of similar form and size, built up with clay and stones, is the usual fire-place of the Turkish soldier in the field, and it answers every purpose of economy and usefulness. The daily issue of fuel to the soldier is three hundred drachms (about thirty-four ounces) of wood, and the same amount of charcoal. This is a much more liberal ration than that which is allowed to the British soldier. The daily ration of fuel for each man in our own army is twenty-four ounces of wood, and twelve ounces of coal.

(*To be continued.*)

---

